# Poly[diaqua­(μ-4,4′-bipyridine-κ^2^ *N*:*N*′)[μ-2,2′-(*p*-phenyl­enedi­oxy)diacetato-κ^2^ *O*:*O*′]cadmium]

**DOI:** 10.1107/S1600536811031400

**Published:** 2011-08-17

**Authors:** Guang-Yin Wang

**Affiliations:** a Department of Chemistry, Dezhou University, Dezhou, Shandong 253023, People’s Republic of China

## Abstract

In the title compound, [Cd(C_10_H_8_O_6_)(C_10_H_8_N_2_)(H_2_O)_2_]_*n*_, the Cd^II^ ion has inversion symmetry and is coordinated by O atoms from two water mol­ecules and two bridging 2,2′-(μ-*p*-phenyl­enedi­oxy)diacetate ligands and two N atoms from two 4,4′-bipyridine ligands, giving a slightly distorted octa­hedral geometry. The diacetate and 4,4′-bipyridine ligands also lie across inversion centers. The bridging ligands form layers parallel to (11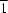), with adjacent layers inter­connected *via* O—H⋯O hydrogen bonds between the coordinated water mol­ecules and the carboxyl­ate O atoms, giving a three-dimensional supra­molecular architecture.

## Related literature

Benzene-1,4-di­oxy­diacetic acid is often used to construct coordination polymers owing to the flexibility of the two phen­oxy­acetate groups, see: Gong *et al.* (2010 ▶); Li *et al.* (2010 ▶); Zhang & Li (2010 ▶).
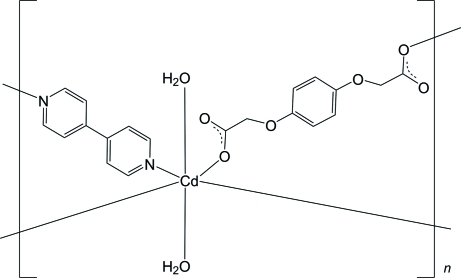


## Experimental

### 

#### Crystal data

[Cd(C_10_H_8_O_6_)(C_10_H_8_N_2_)(H_2_O)_2_]
*M* *_r_* = 528.78
Triclinic, 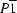

*a* = 5.8612 (6) Å
*b* = 8.2313 (8) Å
*c* = 10.8659 (11) Å
α = 105.640 (1)°
β = 97.6785 (12)°
γ = 97.931 (1)°
*V* = 491.91 (9) Å^3^
*Z* = 1
Mo *K*α radiation
μ = 1.16 mm^−1^
*T* = 296 K
0.21 × 0.11 × 0.04 mm

#### Data collection

Bruker APEXII CCD area-detector diffractometer
Absorption correction: multi-scan (*SADABS*; Bruker, 2001 ▶) *T*_min_ = 0.789, *T*_max_ = 0.956
2533 measured reflections
1691 independent reflections
1667 reflections with *I* > 2σ(*I*)
*R*_int_ = 0.014

#### Refinement

*R*[*F* ^2^ > 2σ(*F* ^2^)] = 0.027
*wR*(*F* ^2^) = 0.067
*S* = 1.14
1691 reflections
142 parameters
H-atom parameters constrained
Δρ_max_ = 0.45 e Å^−3^
Δρ_min_ = −0.39 e Å^−3^

### 

Data collection: *APEX2* (Bruker, 2007 ▶); cell refinement: *SAINT* (Bruker, 2007 ▶); data reduction: *SAINT*; program(s) used to solve structure: *SIR97* (Altomare *et al.*, 1999 ▶); program(s) used to refine structure: *SHELXL97* (Sheldrick, 2008 ▶); molecular graphics: *SHELXTL* (Sheldrick, 2008 ▶); software used to prepare material for publication: *WinGX* (Farrugia, 1999 ▶).

## Supplementary Material

Crystal structure: contains datablock(s) global, I. DOI: 10.1107/S1600536811031400/zs2132sup1.cif

Structure factors: contains datablock(s) I. DOI: 10.1107/S1600536811031400/zs2132Isup2.hkl

Additional supplementary materials:  crystallographic information; 3D view; checkCIF report

## Figures and Tables

**Table 1:** Hydrogen-bond geometry (Å, °)

*D*—H⋯*A*	*D*—H	H⋯*A*	*D*⋯*A*	*D*—H⋯*A*
O1*W*—H2*W*⋯O2^i^	0.85	1.81	2.636 (4)	165
O1*W*—H1*W*⋯O1^ii^	0.85	2.05	2.858 (4)	159

Symmetry codes: (i) 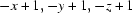; (ii) 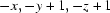.

**Fig. 1.**
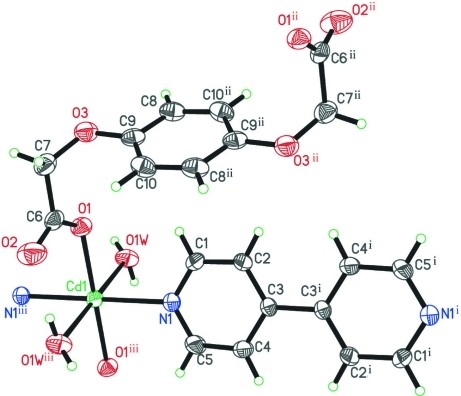
Anisotropic displacement ellipsoid plot of (I) at the 50% probability level. H atoms are represented by circles of arbitrary size. Symmetry code: (i)-x + 1, -y, -z; (ii)-x, -y + 1, -z; (iii) -x + 1, -y + 1, -z + 1.

**Fig. 2.**
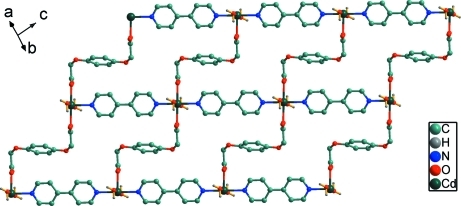
The two-dimensional layered substructure of (I). H atoms are omitted except for those of the water molecules.

**Fig. 3.**
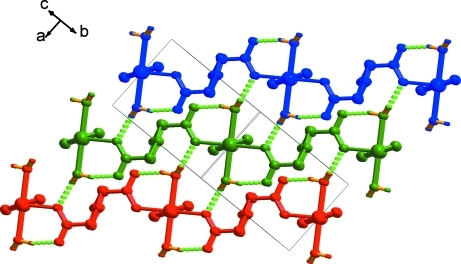
The overall packing diagram of (I) showing showing hydrogen-bonding interactions as dashed lines.

## supplementary crystallographic information

### Comment

Due to the flexibility of the two phenoxyacetate groups, benzene-1,4-dioxydiacetic acid is often used to construct coordination polymers (Zhang & Li, 2010; Gong *et al.*, 2010; Li *et al.*, 2010;). The title coordination polymer [Cd(C_10_H_8_O_6_)(C_10_H_8_N_2_)(H_2_O)_2_]_n_ (I) was obtained under hydrothermal conditions, and its crystal structure is reported here .

The asymmetric unit of (I) is composed of one Cd^II^ cation lying on a crystallographic inversion centre, half a benzene-1,4-dioxydiacetate anion, half a 4,4'-bipyridine molecule and one water molecule. The Cd^II^ ion  is coordinated by O atoms from two water molecules [Cd—O, 2.313 (3)Å] and two from bridging benzene-1,4-dioxydiacetate ligands [Cd—O, 2.253 (2) Å] and two N atoms from two 4,4'-bipyridine ligands [Cd—N, 2.317 (3) Å], giving a slightly distorted octahedral geometry (Fig. 1). The benzene-1,4-dioxydiacetate and 4,4'-bipyridine ligands also lie across inversion centers, with both bridging the Cd^II^ cations to form two-dimensional layers parallel to the (1 1 -1) plane (Fig. 2). These layers are further interconnected *via* O—H···O hydrogen bonds between the coordinated water molecules and the carboxylate O atoms, resulting in a three-dimensional supramolecular architecture (Table 1, Fig. 3).

### Experimental

A mixture of benzene-1,4-dioxydiacetic acid (0.023 g, 0.1 mmol), 4,4'-bipyridine (0.016 g, 0.1 mmol), NaOH (0.008 g, 0.2 mmol) and Cd(NO_3_)_2_. 4H_2_O (0.038 g, 0.1 mmol) in H_2_O (7.0 ml) was placed in a 16 ml Teflon-lined stainless steel vessel and heated to 160 °C for 72 h, then cooled to room temperature at a rate of -5 °C/h. Colorless plate crystals are obtained after filtration.

### Refinement

All H atoms bonded to C atoms were added according to theoretical models, assigned isotropic displacement parameters and allowed to ride on their respective parent atoms [C—H = 0.93–0.97Å and *U*_iso_(H) = 1.2*U*_eq_(C)]. The H atoms attached to O atoms of the water were located from the Fourier map with the O—H distances being fixed at 0.85Å and allowed to ride on their parent oxygen atoms in the final cycles of refinement, with *U*_iso_(H) = 1.2*U*_eq_(O).

### Crystal data

**Table:**

[Cd(C_10_H_8_O_6_)(C_10_H_8_N_2_)(H_2_O)_2_]	*Z* = 1
*M**_r_* = 528.78	*F*(000) = 266
Triclinic, *P*1	*D*_x_ = 1.785 Mg m^−^^3^
Hall symbol: -P 1	Mo *K*α radiation, λ = 0.71073 Å
*a* = 5.8612 (6) Å	Cell parameters from 1861 reflections
*b* = 8.2313 (8) Å	θ = 2.6–27.8°
*c* = 10.8659 (11) Å	µ = 1.16 mm^−^^1^
α = 105.640 (1)°	*T* = 296 K
β = 97.6785 (12)°	Plate, colorless
γ = 97.931 (1)°	0.21 × 0.11 × 0.04 mm
*V* = 491.91 (9) Å^3^	

### Data collection

**Table:**

Bruker APEXII CCD area-detector diffractometer	1691 independent reflections
Radiation source: fine-focus sealed tube	1667 reflections with *I* > 2σ(*I*)
graphite	*R*_int_ = 0.014
φ and ω scans	θ_max_ = 25.0°, θ_min_ = 2.0°
Absorption correction: multi-scan (*SADABS*; Bruker, 2001)	*h* = −6→5
*T*_min_ = 0.789, *T*_max_ = 0.956	*k* = −9→9
2533 measured reflections	*l* = −12→12

### Refinement

**Table:**

Refinement on *F*^2^	Primary atom site location: structure-invariant direct methods
Least-squares matrix: full	Secondary atom site location: difference Fourier map
*R*[*F*^2^ > 2σ(*F*^2^)] = 0.027	Hydrogen site location: inferred from neighbouring sites
*wR*(*F*^2^) = 0.067	H-atom parameters constrained
*S* = 1.14	*w* = 1/[σ^2^(*F*_o_^2^) + (0.0276*P*)^2^ + 0.6011*P*] where *P* = (*F*_o_^2^ + 2*F*_c_^2^)/3
1691 reflections	(Δ/σ)_max_ < 0.001
142 parameters	Δρ_max_ = 0.45 e Å^−^^3^
0 restraints	Δρ_min_ = −0.39 e Å^−^^3^

### Special details

**Table:**

Geometry. All e.s.d.'s (except the e.s.d. in the dihedral angle between two l.s. planes) are estimated using the full covariance matrix. The cell e.s.d.'s are taken into account individually in the estimation of e.s.d.'s in distances, angles and torsion angles; correlations between e.s.d.'s in cell parameters are only used when they are defined by crystal symmetry. An approximate (isotropic) treatment of cell e.s.d.'s is used for estimating e.s.d.'s involving l.s. planes.
Refinement. Refinement of *F*^2^ against ALL reflections. The weighted *R*-factor *wR* and goodness of fit *S* are based on *F*^2^, conventional *R*-factors *R* are based on *F*, with *F* set to zero for negative *F*^2^. The threshold expression of *F*^2^ > σ(*F*^2^) is used only for calculating *R*-factors(gt) *etc*. and is not relevant to the choice of reflections for refinement. *R*-factors based on *F*^2^ are statistically about twice as large as those based on *F*, and *R*- factors based on ALL data will be even larger.

### Fractional atomic coordinates and isotropic or equivalent isotropic displacement parameters (Å^2^)

**Table:**

	*x*	*y*	*z*	*U*_iso_*/*U*_eq_	
C1	0.3288 (6)	0.2750 (4)	0.2007 (3)	0.0314 (7)	
H1	0.2097	0.3389	0.2098	0.038*	
C2	0.3250 (6)	0.1600 (4)	0.0818 (3)	0.0313 (7)	
H2	0.2047	0.1479	0.0130	0.038*	
C3	0.4983 (5)	0.0624 (4)	0.0637 (3)	0.0221 (6)	
C4	0.6685 (6)	0.0859 (5)	0.1715 (3)	0.0371 (8)	
H4	0.7881	0.0223	0.1656	0.044*	
C5	0.6607 (6)	0.2037 (5)	0.2877 (3)	0.0386 (8)	
H5	0.7771	0.2169	0.3586	0.046*	
C6	0.3586 (6)	0.7739 (4)	0.3662 (3)	0.0294 (7)	
C7	0.1802 (6)	0.8591 (4)	0.3031 (3)	0.0358 (8)	
H7A	0.1348	0.9458	0.3706	0.043*	
H7B	0.2542	0.9168	0.2478	0.043*	
C8	−0.1959 (6)	0.4972 (5)	0.0564 (3)	0.0334 (8)	
H8	−0.3285	0.4941	0.0948	0.040*	
C9	−0.0019 (6)	0.6241 (4)	0.1162 (3)	0.0296 (7)	
C10	0.1962 (6)	0.6247 (4)	0.0590 (3)	0.0336 (8)	
H10	0.3294	0.7076	0.0986	0.040*	
N1	0.4958 (5)	0.2990 (3)	0.3034 (2)	0.0271 (6)	
O1	0.2781 (4)	0.6403 (3)	0.3942 (2)	0.0314 (5)	
O2	0.5658 (5)	0.8448 (3)	0.3879 (3)	0.0511 (7)	
O3	−0.0252 (4)	0.7435 (3)	0.2275 (2)	0.0362 (5)	
O1W	0.1584 (4)	0.3373 (3)	0.5183 (2)	0.0385 (6)	
H1W	0.0509	0.3664	0.5598	0.046*	
H2W	0.2264	0.2728	0.5543	0.046*	
Cd1	0.5000	0.5000	0.5000	0.02535 (13)	

### Atomic displacement parameters (Å^2^)

**Table:**

	*U*^11^	*U*^22^	*U*^33^	*U*^12^	*U*^13^	*U*^23^
C1	0.0336 (18)	0.0297 (17)	0.0304 (17)	0.0152 (14)	0.0023 (14)	0.0051 (14)
C2	0.0350 (19)	0.0312 (17)	0.0244 (16)	0.0121 (14)	−0.0063 (13)	0.0049 (13)
C3	0.0242 (16)	0.0216 (14)	0.0195 (15)	0.0021 (12)	0.0033 (12)	0.0057 (12)
C4	0.0302 (18)	0.050 (2)	0.0268 (17)	0.0192 (16)	−0.0002 (14)	−0.0001 (15)
C5	0.035 (2)	0.053 (2)	0.0215 (16)	0.0162 (17)	−0.0027 (14)	0.0008 (15)
C6	0.0330 (19)	0.0304 (17)	0.0256 (16)	0.0132 (15)	0.0093 (14)	0.0038 (13)
C7	0.042 (2)	0.0319 (18)	0.0371 (19)	0.0124 (16)	0.0072 (16)	0.0128 (15)
C8	0.0250 (17)	0.048 (2)	0.0336 (18)	0.0095 (15)	0.0092 (14)	0.0195 (16)
C9	0.0302 (18)	0.0369 (18)	0.0292 (17)	0.0125 (15)	0.0055 (14)	0.0187 (14)
C10	0.0268 (17)	0.0386 (18)	0.0363 (19)	−0.0002 (14)	0.0025 (14)	0.0170 (15)
N1	0.0279 (14)	0.0280 (13)	0.0237 (13)	0.0062 (11)	0.0038 (11)	0.0043 (11)
O1	0.0348 (13)	0.0325 (12)	0.0306 (12)	0.0111 (10)	0.0048 (10)	0.0136 (10)
O2	0.0339 (15)	0.0488 (16)	0.078 (2)	0.0089 (12)	0.0097 (13)	0.0296 (15)
O3	0.0324 (13)	0.0453 (14)	0.0327 (12)	0.0138 (11)	0.0062 (10)	0.0108 (11)
O1W	0.0268 (13)	0.0500 (15)	0.0409 (14)	0.0076 (11)	0.0066 (10)	0.0164 (12)
Cd1	0.0268 (2)	0.02717 (19)	0.02091 (18)	0.00758 (13)	0.00313 (12)	0.00441 (13)

### Geometric parameters (Å, °)

**Table:**

C1—N1	1.334 (4)	C7—H7B	0.9700
C1—C2	1.376 (5)	C8—C10^ii^	1.379 (5)
C1—H1	0.9300	C8—C9	1.384 (5)
C2—C3	1.382 (4)	C8—H8	0.9300
C2—H2	0.9300	C9—O3	1.374 (4)
C3—C4	1.385 (4)	C9—C10	1.388 (5)
C3—C3^i^	1.489 (6)	C10—C8^ii^	1.379 (5)
C4—C5	1.379 (5)	C10—H10	0.9300
C4—H4	0.9300	N1—Cd1	2.317 (3)
C5—N1	1.327 (4)	O1—Cd1	2.253 (2)
C5—H5	0.9300	O1W—Cd1	2.313 (3)
C6—O2	1.234 (4)	O1W—H1W	0.8467
C6—O1	1.265 (4)	O1W—H2W	0.8499
C6—C7	1.526 (5)	Cd1—O1^iii^	2.253 (2)
C7—O3	1.424 (4)	Cd1—O1W^iii^	2.313 (3)
C7—H7A	0.9700	Cd1—N1^iii^	2.317 (3)
			
N1—C1—C2	122.9 (3)	O3—C9—C10	125.2 (3)
N1—C1—H1	118.5	C8—C9—C10	118.7 (3)
C2—C1—H1	118.5	C8^ii^—C10—C9	120.2 (3)
C1—C2—C3	120.6 (3)	C8^ii^—C10—H10	119.9
C1—C2—H2	119.7	C9—C10—H10	119.9
C3—C2—H2	119.7	C5—N1—C1	117.0 (3)
C2—C3—C4	116.1 (3)	C5—N1—Cd1	121.5 (2)
C2—C3—C3^i^	122.3 (3)	C1—N1—Cd1	121.6 (2)
C4—C3—C3^i^	121.6 (3)	C6—O1—Cd1	123.8 (2)
C5—C4—C3	120.0 (3)	C9—O3—C7	117.8 (3)
C5—C4—H4	120.0	Cd1—O1W—H1W	130.3
C3—C4—H4	120.0	Cd1—O1W—H2W	95.3
N1—C5—C4	123.4 (3)	H1W—O1W—H2W	107.2
N1—C5—H5	118.3	O1^iii^—Cd1—O1	180.000 (1)
C4—C5—H5	118.3	O1^iii^—Cd1—O1W	91.72 (10)
O2—C6—O1	126.7 (3)	O1—Cd1—O1W	88.28 (10)
O2—C6—C7	116.8 (3)	O1^iii^—Cd1—O1W^iii^	88.28 (10)
O1—C6—C7	116.5 (3)	O1—Cd1—O1W^iii^	91.72 (10)
O3—C7—C6	114.1 (3)	O1W—Cd1—O1W^iii^	180.0
O3—C7—H7A	108.7	O1^iii^—Cd1—N1	90.66 (9)
C6—C7—H7A	108.7	O1—Cd1—N1	89.34 (9)
O3—C7—H7B	108.7	O1W—Cd1—N1	88.66 (9)
C6—C7—H7B	108.7	O1W^iii^—Cd1—N1	91.34 (9)
H7A—C7—H7B	107.6	O1^iii^—Cd1—N1^iii^	89.34 (9)
C10^ii^—C8—C9	121.2 (3)	O1—Cd1—N1^iii^	90.66 (9)
C10^ii^—C8—H8	119.4	O1W—Cd1—N1^iii^	91.34 (9)
C9—C8—H8	119.4	O1W^iii^—Cd1—N1^iii^	88.66 (9)
O3—C9—C8	116.2 (3)	N1—Cd1—N1^iii^	180.000 (1)
			
N1—C1—C2—C3	0.0 (5)	C7—C6—O1—Cd1	−175.7 (2)
C1—C2—C3—C4	1.1 (5)	C8—C9—O3—C7	170.1 (3)
C1—C2—C3—C3^i^	−179.4 (3)	C10—C9—O3—C7	−11.8 (4)
C2—C3—C4—C5	−1.1 (5)	C6—C7—O3—C9	−66.8 (4)
C3^i^—C3—C4—C5	179.5 (4)	C6—O1—Cd1—O1W	170.5 (2)
C3—C4—C5—N1	−0.1 (6)	C6—O1—Cd1—O1W^iii^	−9.5 (2)
O2—C6—C7—O3	152.4 (3)	C6—O1—Cd1—N1	−100.8 (2)
O1—C6—C7—O3	−29.5 (4)	C6—O1—Cd1—N1^iii^	79.2 (2)
C10^ii^—C8—C9—O3	177.0 (3)	C5—N1—Cd1—O1^iii^	−22.8 (3)
C10^ii^—C8—C9—C10	−1.3 (5)	C1—N1—Cd1—O1^iii^	157.9 (3)
O3—C9—C10—C8^ii^	−176.8 (3)	C5—N1—Cd1—O1	157.2 (3)
C8—C9—C10—C8^ii^	1.3 (5)	C1—N1—Cd1—O1	−22.1 (3)
C4—C5—N1—C1	1.2 (5)	C5—N1—Cd1—O1W	−114.5 (3)
C4—C5—N1—Cd1	−178.2 (3)	C1—N1—Cd1—O1W	66.2 (3)
C2—C1—N1—C5	−1.1 (5)	C5—N1—Cd1—O1W^iii^	65.5 (3)
C2—C1—N1—Cd1	178.2 (3)	C1—N1—Cd1—O1W^iii^	−113.8 (3)
O2—C6—O1—Cd1	2.1 (5)		

Symmetry codes: (i) −*x*+1, −*y*, −*z*; (ii) −*x*, −*y*+1, −*z*; (iii) −*x*+1, −*y*+1, −*z*+1.

### Hydrogen-bond geometry (Å, °)

**Table:**

*D*—H···*A*	*D*—H	H···*A*	*D*···*A*	*D*—H···*A*
O1W—H2W···O2^iii^	0.85	1.81	2.636 (4)	165
O1W—H1W···O1^iv^	0.85	2.05	2.858 (4)	159

Symmetry codes: (iii) −*x*+1, −*y*+1, −*z*+1; (iv) −*x*, −*y*+1, −*z*+1.
